# Association of a null allele of *SPRN* with variant Creutzfeldt–Jakob disease

**DOI:** 10.1136/jmg.2008.061804

**Published:** 2008-09-17

**Authors:** J A Beck, T A Campbell, G Adamson, M Poulter, J B Uphill, E Molou, J Collinge, S Mead

**Affiliations:** MRC Prion Unit and Department of Neurodegenerative Disease, UCL Institute of Neurology, London, UK

## Abstract

**Background:**

No susceptibility genes have been identified in human prion disase, apart from the prion protein gene (*PRNP*). The gene *SPRN*, encodes Shadoo (Sho, shadow of prion protein) which has protein homology and possible functional links with the prion protein.

**Methods:**

A genetic screen was carried out of the open reading frame of *SPRN* by direct sequencing in 522 patients with prion disease, including 107 with variant Creutzfeldt–Jakob disease (vCJD), and 861 healthy controls.

**Results:**

A common coding variant of *SPRN*, two further single nucleotide polymorphisms (SNPs) and three rare insertion or deletion variants were found. A single base-pair insertion at codon 46, predicted to cause a frameshift and potentially a novel protein, was found in two patients with vCJD but not in controls (p = 0.01). Two linked SNPs, one in intron 1 and the other a missense variant at codon 7, were associated with risk of sporadic CJD (p = 0.009).

**Conclusion:**

These data justify the functional genetic characterisation of *SPRN* and support the involvement of Shadoo in prion pathobiology.

Despite clear evidence from mouse linkage studies of multiple genetic loci affecting incubation periods of prion diseases,^1^ ^2^ no specific human genes have been identified, apart from the prion protein gene (*PRNP*). A recently characterised highly conserved gene, *SPRN*, encodes Shadoo (Sho, shadow of prion protein) which has protein homology and possible functional links with the prion protein.^3^

Human prion diseases are a phenotypically diverse range of fatal neurodegenerative disorders that are unique in having sporadic, acquired and inherited aetiologies.^4^ Common to the pathogenesis of all prion diseases is a central role for the autocatalytic misfolding of the prion protein (PrP).^5^ These uncommon neurodegenerative diseases have come under close scrutiny in recent years because of a public health threat related to the epizootic of bovine spongiform encephalopathy (BSE), its human counterpart variant Creutzfeldt–Jakob disease (vCJD)^6^^–^8 and the subsequent secondary transmission of vCJD by blood transfusion.^9^ Sporadic forms of human prion disease account for approximately 85% of total prion disease cases, most typically appearing as a rapidly progressive multifocal dementia with myoclonus (sCJD). Multiple distinct prion strains in sporadic disease associate with a variety of clinicopathological phenotypes.^10^ Conversely, vCJD is associated with a distinct and unique prion strain, and has been described to date in around 200 patients worldwide with most (>160 cases) occurring in the UK. As a group, patients with vCJD differ from those with sCJD in their relatively early age of onset and longer duration, prominent psychiatric and sensory features at presentation, and characteristic neuropathology and prominent lymphoreticular prion infection.^11^

Although there is only one known familial concurrence of vCJD, there is a strong likelihood that genetic susceptibility has played a part in determining why particular individuals succumbed following the widespread population exposure to dietary BSE prions. Epidemiological case control studies have shown no clear-cut evidence for unusual dietary or occupational exposure.^12^ *PRNP* is polymorphic in Europeans at codon 129, between methionine (∼60% allele frequency) and valine. All clinical vCJD cases examined have been homozygous for methionine,^13^ representing the strongest association of a common genotype with any disease. Support for the existence of novel genetic factors is derived from mouse quantitative locus studies, which have shown multiple regions, unlinked to *Prnp*, that control the incubation time to BSE prions.^1^ ^14^ ^15^

The normal function of PrP, its molecular interactions in the healthy cell, and its pathogenesis and neurotoxic pathways remain unclear. Proteins encoded by genetic loci that modify susceptibility to, or incubation time in, prion disease might do so by involvement with the normal functional pathways of PrP, either as a ligand or homologue, and several putative PrP ligands have been proposed.^16^ The recently characterised prion protein family,^17^ including Doppel^18^ and Shadoo,^19^ are pre-eminent candidate functional homologues of PrP, but several lines of evidence contradict involvement of *PRND* genetic variation and Dpl in human prion disease.^17^ ^20^ ^21^

*SPRN*, the gene encoding Sho, was identified by comparative gene analysis, and this gene and its encoded protein have several characteristics suggestive of a role in prion biology.^19^ The *SPRN* gene comprises two exons, the latter containing the entire open reading frame (ORF). Also in common with *PRNP*, high levels of conservation of this gene exist between many species from fish to mammals,^22^ with highest levels of *SPRN* conservation present in its hydrophobic domain. In addition, both proteins are predicted to have glycophosphotidylinositol (GPI) anchor attachment, a single transmembrane domain and a highly conserved N-terminal sequence. Recent work has shown overlapping expression patterns of PrP and Sho in the CNS, the similarity of Sho transgenes to PrP in their ability to counteract the neurotoxic effects of either Doppel or aminoterminal truncated PrP, and reduced levels of Sho in experimental prion infection. It has therefore been hypothesised that Sho may interact either directly or indirectly with PrP or its ligand.^3^ In this study, we provide new evidence of a role for *SPRN* in prion pathobiology by showing that Sho variants are associated with two human prion diseases.

## METHODS

Ethics approval was obtained from the UCL Institute of Neurology and National Hospital for Neurology and Neurosurgery Local Research Ethics Committee, and all participants gave informed consent.

The study comprised patients with sCJD, patients with vCJD and a control group. The sCJD group comprised 415 patients, who were definite or probable cases of sCJD according to World Health Organization (WHO) diagnostic criteria. The 107 definite or probable patients with vCJD studied also met WHO diagnostic criteria and were diagnosed either by tonsillar biopsy, post-mortem analysis of brain tissue, or established clinical criteria and imaging findings. The control cohort consisted of 92 healthy UK controls matched for age and sex to the patients with vCJD, 137 UK healthy blood donor controls, 446 UK healthy controls from the European Collection of Cell Cultures (ECACC) collection, and 186 unrelated healthy control individuals from the Centre d’Etude du Polymorphisme Humain (CEPH) collection. 

We performed direct gene resequencing on peripheral blood samples from cases and controls to investigate the possibility of a significant association between genetic variation within the *SPRN* ORF and susceptibility to sCJD or vCJD. Genomic DNA was isolated using a commercial extraction kit (Nucleon BACC2; GE Healthcare UK, Little Chalfont, Buckinghamshire, UK) following the supplied protocol. DNA concentrations were determined using a spectrophotometer (Nanodrop ND-1000; Thermo Fisher Scientific, Waltham, Massachusetts, UK), and diluted in Tris/EDTA buffer in aliquots of 20 ng/μl to prepare 96-well plates for PCR.

For DNA sequencing, oligonucleotide primers were designed to amplify two overlapping amplicons to span the entire ORF and were designed using DNA sequence from reference contig accession number AL161645 (exact location of primers with reference to AL161645 are given in parentheses after each primer described below). Amplicon 1 was 359 bp (−100 bp to +259 bp) and amplified with sense 5′-CCTCTGCTCTCCAGCCTTG-3′ (8404-8422) and antisense 5′-AGCCCGCCGCCAGGCC-3′ (8064–8080), and amplicon 2 was 739 bp (+48 bp to +786 bp) and amplified with sense 5′-CTTCCTCTGCGACAGCGGCG-3′ (8257–8276) and antisense 5′-GCGATGGAGCGTGGCTGGG-3′ (7566–7585). PCR reactions containing a final concentration of 1 mol/l betaine were thermally cycled with an initial denaturation step at 95°C for 5 minutes, followed by 35 cycles of 95°C for 30 s, 65.5°C (amplicon 1) or 63.1°C (amplicon 2) for 30 s and 72°C for 45 s, and then a final extension step at 72°C for 5 minutes. Cleaned PCR products were sequenced in reactions including betaine 1 mol/l (final concentration) and separated by electophoresis on an automated sequencer (3130xl; Applied Biosystems, Foster City, California, USA). Data analysis was performed using Seqscape software V.2.5 (Applied Biosystems) with analysis filter settings adjusted to allow assembly of poor data due to insertions of deletions (maximum mixed bases 95%, maximum uncalled bases 95%, minimum clear length of 1 bp, and minimum sample score of 1). Poor data or failed reactions were removed from projects by visual inspection. To assess the expression level of the frameshift allele (p.Ala46GlyfsX294) in postmortem frontal cortex, total RNA was extracted from 100 mg of frozen tissue using (TRIzol Plus Purification kit; Invitrogen, Paisley, UK). A commercial kit (Omniscript Reverse Transcription Kit; Qiagen, Crawley, West Sussex, UK) and random hexamers (Invitrogen) were used to produce cDNA. Generation of a fluorescently labelled PCR product of both genomic DNA (gDNA) and cDNA was performed in both case and controls (suitable case material was available from only one patient with vCJD) using sense 5′-FAM-AGGTGCGCGGGGCAGT-3′ (8296–8311) and antisense 5′-CTCACGCGCACCCTCGA-3′ (8348–8364) primers. The resultant 69 bp amplicon was separated by electrophoresis on an automated sequencer (3130xl; Applied Biosystems) with LIZ500 size standard and peak size, height and area from both gDNA and cDNA were calculated using GeneMapper software V.4.0 (Applied Biosystems).

### Statistical analysis

Statistical and genetic analyses were performed using Haploview,^23^ PLINK (http://pngu.mgh.harvard.edu/∼purcell/plink/index.shtml) and SSPS software. For sCJD, the analysis was 80% powered to detect a heterozygous genotype relative risk of 1.25 in a multiplicative model. Haplotype frequencies were estimated with an expectation–maximisation (EM) algorithm.

## RESULTS

Six different genetic variations were detected in the 2766 chromosomes sequenced (table 1). Of these, three were of an insertion or deletion (indel) variety, two were non-coding single-nucleotide polymorphisms (SNPs) either inside or outside the *SPRN* ORF, and one was a coding SNP. This common missense variant was found in the *SPRN* signal peptide sequence (c.20C→T, p.T7M), and is linked to a G→A transversion 11 bp 5′ of the *SPRN* translation start methionine. We found 5′-11A linked to p.7M and 5′-11G linked to p.7T. These alleles form the basis of derived haplotypes designated *1 and *2 respectively (table 1). Within this haplotype scheme, the *1 related haplotypes were all associated with c.20T and c.183C of the *SPRN* ORF, whereas the *2 related haplotypes were associated with c.20C and either position c.183C (haplotypes *2A and *2C) or c.183T (haplotypes *2 and *2C). A 12 bp deletion variation was also detected in four healthy controls (three on the *1 haplotype and one on the *2 haplotype), corresponding to the in-frame deletion of an AAAG amino acid repeat motif located in the highly conserved hydrophobic central domain of Sho, reducing the polypeptide chain to 147aa from the most commonly observed length of 151aa. These variants were designated haplotypes *1A and *2C respectively. A second similar indel variation was detected in a single CEPH control, and involved a 12 bp insertion in the hydrophobic domain resulting in an additional AAAG amino acid repeat motif, increasing the full length protein to 155aa, and was designated haplotype *1B. The third indel variation detected in two patients with vCJD only was an insertion of a single guanine base pair at codon 46. This frameshift mutation creates a potential extended ORF of 882 bp, designated haplotype *2B. Positive results of statistical analysis between genotypes and alleles of cases versus controls are shown in table 2.

**Table 1:** Genetic variants found in the study

Variation		Key	Allele counts			Haplotypes and frequency						
DNA	Protein		vCJD (n = 107)	sCJD (n = 415)	HC (n = 861)	*1	*1A	*1B	*2	*2A	*2B	*2C
5′-11A→G	–	A:G	152:62	598:232	1155:567	A	A	A	G	G	G	G
c.20C→T	p.Thr7Met	T:C	152:62	598:232	1155:567	T	T	T	C	C	C	C
c.136_137insG	p.Ala46GlyfsX294	wt:ins	212:2	830:0	1722:0	wt	wt	wt	wt	wt	ins	wt
c.183C→T	–	C:T	171:43	672:158	1344:378	C	C	C	T	C	T	C
c.216_227delAGC CGGGGCGGC	p.Ala72_Ala75del	wt:del	214:0	830:0	1718:4	wt	del§	wt	wt	wt	wt	del§
c.239_240insGGC GGGAGCGGC	p.Ala79_Ala80ins AlaAlaGlyAla	wt:ins	214:0	830:0	1721:1	wt	wt	ins	wt	wt	wt	wt
			vCJD			152† (71)	0†	0†	41† (19)	19† (9)	2† (1)	0†
				sCJD		598‡ (72)	0‡	0‡	158‡ (19)	74‡ (9)	0‡	0‡
					Control	1151§ (67)	3§ (<1)	1§ (<1)	379§ (22)	187§ (11)	0§	1§ (<1)

del, Deletion; HC, healthy controls; ins, insertion; sCJD, sporadic Creutzfeldt–Jakob disease; vCJD, variant Creutzfeldt–Jakob disease; wt, wild type.

†vCJD.

‡sCJD.

§Healthy controls.

Individual haplotypes and their frequencies, given as counts with percentages in parentheses, are displayed vertically, calculated by expectation-maximisation algorithm (Haploview).

Alleles and allele counts, together with a key to the allele counts, are displayed horizontally.

Insertion and deletion alleles are coded as wild type (wt) or ‘ins/del’ depending on the absence or presence of the indel variation compared with contig AL161645, respectively.

**Table 2:** Results from tests performed using genotypic and allelic (trend) models

Variation	MAF		Model					
			Genotypic			Allelic (trend)		
			Counts	χ^2^	p Value	Counts	χ^2^	p Value
5′-11A→G/c.20T→C	0.29	vCJD	11/40/56	3.655	0.16	62/152	1.43	0.23
	0.28	sCJD	30/172/213	7.135	**0.028**	232/598	6.8	**0.009**
	0.33	Control	81/405/375	–	–	567/1155	–	–
c.136_137insG	0.01	vCJD	0/2/105	NA	NA	2/212	16.1	**0.01†**
	0	sCJD	0/0/415	NA	NA	0/830	NA	NA
	0	Control	0/0/861	–	–	0/1722	–	–

NA, not applicable; sCJD, sporadic Creutzfeldt–Jakob disease; vCJD, variant Creutzfeldt–Jakob disease.

†Denotes Fisher Exact test applied because cell counts were small. Genotypic counts and allelic counts are displayed AA/AB/BB and A/B respectively, where A = 5′-11G (c.20C) or c.136_137insG.

Bold type denotes significance.

The SNPs 5′-11A→G and c.20T→C were found to be in complete linkage disequilibrium and were therefore grouped. A genotypic model gave p = 0.028, and an allelic model gave p = 0.009, comparing sCJD with controls where an increase of 5′-11A and linked c.20T was observed in cases. A similar increase was observed in vCJD cases although the small numbers meant this did not reach significance. An allelic model was used with the Fisher Exact test when analysing the level of significance of the frameshift allele detected in two vCJD cases (p = 0.01, table 2). 

*SPRN* genotype data was also stratified by PRNP codon 129 status (table 3). No significant association was found between control codon 129 status and *SPRN* genotype and therefore control data is shown unstratified. Stratification of sCJD *SPRN* genotype by codon 129 status showed a strongly significant association between c.20T and codon 129 methionine homozygous sCJD (p = 0.007). Further stratification of *SPRN* genotype data was performed on vCJD cases by age at onset of disease, duration and year of onset as a measure of susceptibility within the vCJD group (table 4), but no significant associations with these aspects of phenotype were detected. Finally, sCJD *SPRN* genotype data was stratified by age at onset and duration of disease and PrP^Sc^ type (table 5). Again, no significant associations were detected.

**Table 3:** Stratification of *SPRN* genotypes in patients with sporadic Creutzfeldt–Jakob disease

Genotype c.20	Control	sCJD		
		MM	MV	VV
TT	375	131	50	29
TC	405	98	31	41
CC	81	16	8	8
p Value	–	0.007	0.038	0.67

sCJD, sporadic Creutzfeldt–Jakob disease.

Because *SPRN* SNPs at positions intron 1 5′-11 and c.20 show complete linkage, the genotypes shown are representative of either genotype 5′-11A/5′-11G, or c.20T/c.20C.

**Table 4:** Stratification of phenotypic data in patients with variant Creutzfeldt–Jakob disease

Genotype c.20	Age at disease onset (years)			Duration of disease (months)			Year of onset		
	Mean	SD	Median	Mean	SD	Median	Mean	SD	Median
TT	29.9	12.2	28	16.6	5.8	16	1998.4	2.4	1999
TC	29.6	10.3	25	15.7	5.2	14.5	1999.0	2.4	1999
CC	33.5	9.8	29	19.5	8.4	19	1998.6	2.6	1998

SD, standard deviation.

**Table 5:** Stratification of phenotypic data by PrP^Sc^ (molecular strain) type (according to the London classification^27^), according to *SPRN* genotypes in patients with sporadic Creutzfeldt–Jakob disease

Genotype c.20	Age at disease onset (years)			Duration of disease (months)			PrP^Sc^ type			
							1	2	3	2 + 3
	Mean	SD	Median	Mean	SD	Median				
TT	55.3	21.5	62	9.1	13.6	5	6	33	9	2
CT	52.8	23.3	61	6.6	7.5	4	5	17	11	0
CC	57.3	27.2	68	7.3	4.8	7	1	3	1	0

SD, standard deviation.

Unfortunately, RNA recovered from the single vCJD case possessing the frameshift mutation was of insufficient quality for our assessment of expression level of mutant transcripts. The year of death of the two frameshift mutation patients with vCJD was 1996 and 1995, towards the start of the vCJD epidemic. The clinical history of the patient who died in 1996 and was examined in detail involved depression, behavioural disturbance, altered mood with emotional lability, cognitive decline and progressive chorea of 12 months’ duration. Clinical examination was notable for global rigidity, generalised chorea, a broad-based gait and attentional deficits. This patient died aged 28 years. Fewer details are available for the second patient with vCJD with the frameshift mutation who died aged 29 years.

## DISCUSSION

We report sequencing of the *SPRN* ORF in all available samples of UK patients with vCJD, 415 patients with definite or probable sCJD and 861 healthy controls. We found a significant association between a frameshift mutation and vCJD (p = 0.01) and between c.20T and sCJD (p = 0.009). Three major haplotypes exist in the healthy control sample set tested and are defined by the three SNPs detected: A/T/C, G/C/T, G/C/C (5′-11/c.20/c.183, respectively). Haplotype frequencies were approximately 0.69, 0.21, and 0.10 for these haplotypes, respectively (averaged from cases and controls; table 1). Additional haplotypes exist at low frequency and are associated with indel variations of the hydrophobic core in healthy controls and with a frameshift mutation in two patients with vCJD. Given the low frequency of indel variants discovered in healthy controls, it is not surprising that we did not detect a similar polymorphism in the cases tested.

Our investigations have revealed genetic variants that were discovered in cases only, cases and controls, or controls only. The effect of these variants on Sho biology or prion biology is unknown, but the nature of each and their location within *SPRN* may provide insight into their likely functionality. The indel variations discovered in five healthy controls all affect the size of the hydrophobic core of the Sho protein from the most common size of 23aa to either 19aa or 27aa depending on alleles containing a 12 bp deletion or insertion respectively. Missense mutations of the hydrophobic core of PrP are associated with disease and have been shown to lead to a transmembrane topological variant of PrP with the C-terminus being luminal and the N-terminus cytoplasmic.^24^ The significance of expansion or contraction of the hydrophobic core by insertion or deletion of 4aa in Sho remains unknown, although our finding of such variants in healthy controls suggests toleration of these in Sho.

The allele we report associated with sCJD, for which we also report a trend towards the same allele in vCJD, has two genetic variations that determine haplotype in our scheme. 1* haplotypes are associated with 5′-11A/c.20T (p.7M) and we have shown that these are significantly more common in sCJD. It remains possible that either 5′-11A→G or c.20T→C are functional SNPs, or indeed that an as yet undetermined functional polymorphism exists in unscreened regions of *SPRN*. The SNP 5′-11A→G is located in the short 16 bp untranslated region (UTR) of *SPRN* exon 1 and has a probability of 0.82 that alternative splicing may occur when guanine is present at bp 5′-11 as determined by Netgene2 (http://www.cbs.dtu.dk/services/NetGene2/) analysis. Conversely, no alternative splicing is predicted for 5′-11A. The biological effect of excision of 5 bp of 5′UTR close to the ORF is unknown, and further complexity exists in deciphering the functional element linked to sCJD due to c.20C→T. In this, an increase of the p.7M allele was found in sCJD, and codon 7 is predicted to be contained within the signal peptide sequence of *SPRN*. The frequency of these alleles in healthy controls is strongly suggestive that no deleterious transport of Sho occurs due to methionine at residue 7 of the signal sequence, but this does not obviate the effect of subtle influences on protein transport upon establishment and progression of prion disease. Indeed, in a mouse model exploring the role of mutations in the signal peptide sequence of PrP on hydrophobic tract mutants, it has been shown that the double mutant L9R-3AV resulted in abnormal PrP trafficking.^25^

The effects of our final finding of a significant association of a frameshift mutation with vCJD is more easily rationalised. Insertion of a single guanine at codon 46 of *SPRN* would cause a putative polypeptide chain of 294aa if translation of mutant transcript occurred, and a corresponding reduction in normal Sho protein. Nonsense-mediated mRNA decay (NMD) is a mechanism by which transcripts containing premature translation termination codons are degraded,^26^ and as most frameshift mutations result in the presence of a premature termination codon, most mRNA transcripts containing a frameshift mutation are predicted to be degraded by NMD. Despite our efforts to assess the levels of the p.Ala46GlyfsX294 message from frontal cortex tissue of a single patient with vCJD, owing to prolonged post-mortem delay and several sample freeze–thaw cycles we were unable to show whether or not the transcript exists at all, or whether it exists at reduced or equal measure to the wild-type allele. Nevertheless, the presence of a rare frameshift allele in two patients with vCJD remains of interest, particularly as these patients were amongst the most susceptible patients based on time of onset and were among the first British patients. In all other respects, the phenotype of these two cases was unremarkable within the larger vCJD cohort. The possibility that these two patients, who resided in different parts of the UK, were closely related, was excluded by genome-wide genotype data (500K arrays; Affymetrix, Santa Clara, California, USA; data not shown). More definitive genetic evidence that *SPRN* null alleles cause susceptibility to vCJD would require an allelic series, which we were unable to show with our necessarily small sample size.

Although confirmation of our findings in further non-UK cases of vCJD and sCJD would be extremely valuable, our data support the hypothesis that *SPRN* genetic variants are involved in the pathobiology of prion disease. Interactions between PrP and Sho in health and disease are not yet clear, but these genetic data are consistent with an existing model that proposes a neuroprotective role for Sho.^3^ Our associations of a frameshift mutation, which may lead to a null allele, with vCJD, and *1 *SPRN* haplotypes with sCJD, are consistent with a direct or indirect interaction between proteins in the disease process. However, the genetic evidence is not straightforward in that different alleles were associated with two categories of human prion disease. Whether this finding relates to our necessarily small sample sizes, as the c.20T allele was more common, but not significantly so, in vCJD, or alternatively reflects the distinct pathogenesis of sporadic and vCJD, is unresolved. Further investigations are required to explore these possibilities, and in particular, creation of null or transgenic cellular or mouse models of *SPRN*.

## References

[1] Lloyd S Onwuazor ON Beck J Mallinson G Farrall M Targonski P Collinge J Fisher E Identification of multiple quantitative trait loci linked to prion disease incubation period in mice. Proc Natl Acad Sci USA 2001;98:6279–83 11353827 10.1073/pnas.101130398 PMC33459

[2] Stephenson DA Chiotti K Ebeling C Groth D DeArmond SJ Prusiner SB Carlson GA Quantitative trait loci affecting prion incubation time in mice. Genomics 2000;69:47–53 11013074 10.1006/geno.2000.6320

[3] Watts JC Drisaldi B Ng V Yang J Strome B Horne P Sy MS Yoong L Young R Mastrangelo P Bergeron C Fraser PE Carlson GA Mount HT Schmitt-Ulms G Westaway D The CNS glycoprotein Shadoo has PrP(C)-like protective properties and displays reduced levels in prion infections. EMBO J 2007;26:4038–50 17703189 10.1038/sj.emboj.7601830 PMC1950727

[4] Collinge J Prion diseases of humans and animals: their causes and molecular basis. Annu Rev Neurosci 2001;24:519–50 11283320 10.1146/annurev.neuro.24.1.519

[5] Prusiner SB Novel proteinaceous infectious articles cause scrapie. Science 1982;216:136–44 6801762 10.1126/science.6801762

[6] Collinge J Sidle KCL Meads J Ironside J Hill AF Molecular analysis of prion strain variation and the aetiology of ‘new variant’ CJD. Nature 1996;383:685–690 8878476 10.1038/383685a0

[7] Bruce ME Will RG Ironside JW McConnell I Drummond D Suttie A McCardle L Chree A Hope J Birkett C Cousens S Fraser H Bostock CJ Transmissions to mice indicate that ‘new variant’ CJD is caused by the BSE agent. Nature 1997;389:498–501 9333239 10.1038/39057

[8] Hill AF Desbruslais M Joiner S Sidle KCL Gowland I Collinge J The same prion strain causes vCJD and BSE. Nature 1997;389:448–50 9333232 10.1038/38925

[9] Llewelyn CA Hewitt PE Knight RS Amar K Cousens S Mackenzie J Will RG Possible transmission of variant Creutzfeldt-Jakob disease by blood transfusion. Lancet 2004;363:411–12 14962520 10.1016/S0140-6736(04)15486-X

[10] Wadsworth J Hill AF Beck J Collinge J Molecular and clinical classification of human prion disease. Brit Med Bull 2003;66:241–54 14522862 10.1093/bmb/66.1.241

[11] Spencer MD Knight RS Will RG First hundred cases of variant Creutzfeldt-Jakob disease; restrospective case note review of early psychiatric and neurological features. BMJ 2002;324:1479–82 12077031 10.1136/bmj.324.7352.1479 PMC116442

[12] Ward HJ Everington D Cousens SN Smith-Bathgate B Leitch M Cooper S Heath C Knight RS Smith PG Will RG Risk factors for variant Creutzfeldt-Jakob disease: a case-control study. Ann Neurol 2006;59:111–20 16287153 10.1002/ana.20708

[13] Collinge J Beck J Campbell T Estibeiro K Will RG Prion protein gene analysis in new variant cases of Creutzfeldt-Jakob disease. Lancet 1996;348:56 8691941 10.1016/s0140-6736(05)64378-4

[14] Lloyd S Uphill JB Targonski PV Fisher E Collinge J Identification of genetic loci affecting mouse-adapted bovine spongiform encephalopathy incubation time in mice. Neurogenetics 2002;4:77–81 12481985 10.1007/s10048-002-0133-9

[15] Manolakou K Beaton J McConnell I Farquar C Manson J Hastie ND Bruce M Jackson IJ Genetic and environmental factors modify bovine spongiform encephalopathy incubation period in mice. Proc Natl Acad Sci U S A 2001;98:7402–7 11404459 10.1073/pnas.121172098 PMC34681

[16] Linden R Martins VR Prado MA Cammarota M Izquierdo I Brentani RR Physiology of the prion protein. Physiol Rev 2008;88:673–728 18391177 10.1152/physrev.00007.2007

[17] Watts JC Westaway D The prion protein family: Diversity, rivalry, and dysfunction. Biochim Biophys Acta 2007;1772:654–72 17562432 10.1016/j.bbadis.2007.05.001

[18] Moore RC Lee IY Silverman GL Harrison PM Strome R Heinrich C Karunaratne A Pasternak SH Chishti MA Liang Y Mastrangelo P Wang K Smit AFA Katamine S Carlson GA Cohen FE Prusiner SB Melton DW Tremblay P Hood LE Westaway D Ataxia in prion protein (PrP)-deficient mice is associated with upregulation of the novel PrP-like protein Doppel. *J Mol Biol* 1999;292:797–817 10.1006/jmbi.1999.3108 10525406

[19] Premzl M Sangiorgio L Strumbo B Marshall Graves JA Simonic T Gready JE Shadoo, a new protein highly conserved from fish to mammals and with similarity to prion protein. Gene 2003;314:89–102 14527721 10.1016/s0378-1119(03)00707-8

[20] Peoc’h K Guerin C Brandel JP Launay JM Laplanche JL First report of polymorphisms in the prion-like protein gene (PRND): implications for human prion diseases. Neurosci Lett 2000;286:144–8 10825657 10.1016/s0304-3940(00)01100-9

[21] Mead S Beck J Dickinson A Fisher E Collinge J Examination of the human prion protein-like gene Doppel for genetic susceptibility to sporadic and variant Creutzfeldt-Jakob disease. Neurosci Lett 2000;290:117–20 10936691 10.1016/s0304-3940(00)01319-7

[22] Premzl M Gamulin V Comparative genomic analysis of prion genes. BMC Genomics 2007;1. 10.1186/1471-2164-8-1 PMC1781936 17199895

[23] Barrett JC Fry B Maller J Daly MJ Haploview: analysis and visualization of LD and haplotype maps. Bioinformatics 2005;21:263–5 15297300 10.1093/bioinformatics/bth457

[24] Hegde RS Mastrianni JA Scott MR DeFea KA Tremblay P Torchia M DeArmond SJ Prusiner SB Lingappa VR A transmembrane from of the prion protein in neurodegenerative disease. Science 1998;279:827–34 9452375 10.1126/science.279.5352.827

[25] Stewart RS Harris DA A transmembrane form of the prion protein is localized in the Golgi apparatus of neurons. J Biol Chem 2005;280:15855–64 15671025 10.1074/jbc.M412298200

[26] Nagy E Maquat LE A rule for termination-codon position within intron-containing genes: when nonsense affects RNA abundance. Trends Biochem Sci 1998;23:198–9 9644970 10.1016/s0968-0004(98)01208-0

[27] Hill AF Joiner S Wadsworth J Sidle KC Bell JE Budka H Ironside JW Collinge J Molecular classification of sporadic Creutzfeldt-Jakob disease. Brain 2003;126:1333–46 12764055 10.1093/brain/awg125

